# Synapse-associated protein 102 – a highly mobile MAGUK predominate in early synaptogenesis

**DOI:** 10.3389/fnmol.2023.1286134

**Published:** 2023-10-19

**Authors:** Dominique Alexandra De Los Reyes, Mohammad Yaman Karkoutly, Yonghong Zhang

**Affiliations:** School of Integrative Biological and Chemical Sciences, The University of Texas Rio Grande Valley, Edinburg, TX, United States

**Keywords:** synapse-associated protein 102, MAGUK family scaffolding proteins, early synaptogenesis, synaptic mobility, postsynaptic density regulation, glutamate receptors

## Abstract

Neurodevelopmental and neurodegenerative disorders are primarily characterized by serious structural and functional changes in excitatory glutamatergic synapses in the brain, resulting in many synaptic deficits and aberrant synapse loss. It is a big challenge to reverse these synaptic impairments as a treatment for neurological diseases in the field. Extensive research on glutamate receptors as therapeutic targets has been done but with little success shown in human trials. PSD-95-like MAGUK proteins perform a pivotal role in regulating the trafficking and stability of glutamate receptors that are important to postsynaptic structure and function. MAGUK and MAGUK-modulated synaptic pathways are becoming promising candidates for developing therapeutic targets. As a MAGUK protein, SAP102 is not understood well compared to PSD-95. Here, we review the current research on SAP102 including its synaptic functions and regulation, especially its expression and functions in the early stage of synaptogenesis and the association with neurodevelopmental disorders. This review presents valuable information for future structural and functional studies of SAP102 to reveal its roles in young and mature neurons. It provides clues for developing potential remedies to reverse synaptic impairments and strategies to grow new neurons.

## Introduction

Neurodevelopmental and neurodegenerative disorders are primarily characterized by serious structural and functional changes in excitatory glutamatergic synapses in the brain. Reversing synaptic dysfunctions and impairments, such as regeneration or growth of new neurons, is still a major challenge and requires a comprehensive understanding of synaptic structures and dynamic regulatory mechanisms. The postsynaptic density (PSD) is a cytoskeletal specialization of multiprotein signaling complexes associated with postsynaptic membranes, which is crucial for synaptic strength and plasticity in excitatory neurons. Here, the membrane-associated guanylate kinase (MAGUK) protein family, particularly PSD-95, are found playing a central role in scaffolding key PSD components and regulating PSD nanostructure and glutamate receptors essential for synaptic signaling, development, and survival (Won et al., 2017). The MAGUKs include PSD-95, PSD-93, synapse-associated protein 102 (SAP102), and SAP97; they are all associated with neurological disorders (Vyas and Montgomery, 2016). The perturbations to PSD-MAGUKs underlie the synaptic pathology of neurological diseases, therefore they show strong therapeutic promise (Zhou et al., 2015; Su et al., 2018). Among them, PSD-95 has been well-studied structurally and functionally and its defectiveness has been linked to neurodevelopmental disorders (Levy et al., 2022). Other MAGUKs, especially SAP102, remain elusive for their synaptic functions and regulation.

**Figure 1:**
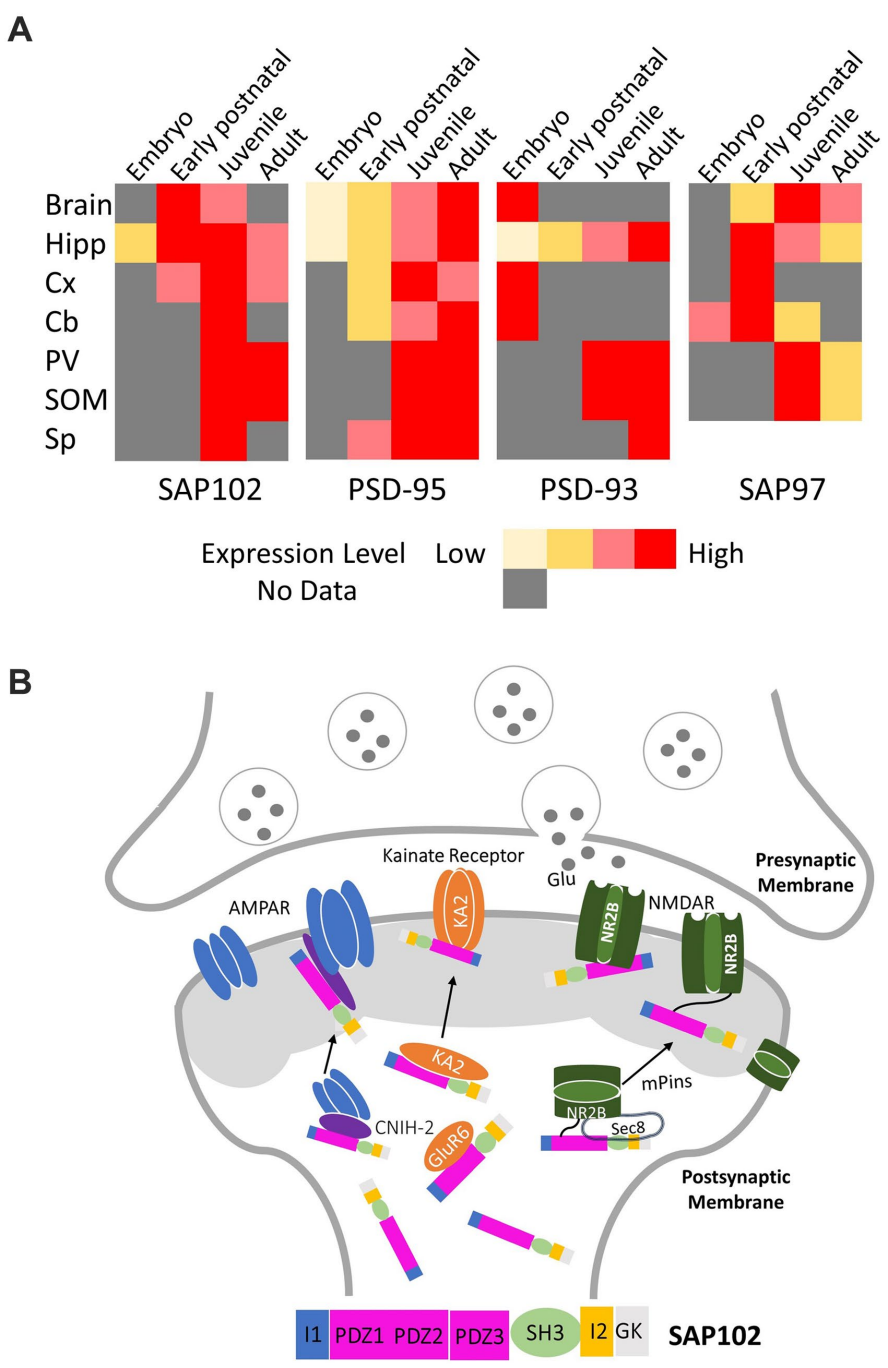
**(A)** Expression pattern of PSD-MAGUKs in mammalian replotted according to the literature (Oliva et al., 2012). The color code is used to indicate the relative expression during development in brain, Hipp (Hippocampus), Cx (Cortex), Cb (Cerebellum), PV (parvalbumin positive interneurons of the visual cortex), SOM (somatostatin positive interneurons of the visual cortex), Sp (spinal cord). **(B)** Representative signaling pathways of SAP102 regulating synaptic trafficking and postsynaptic targeting of glutamate receptors (NADMRs, AMPARs, and kainate receptors).

**Figure 2:**
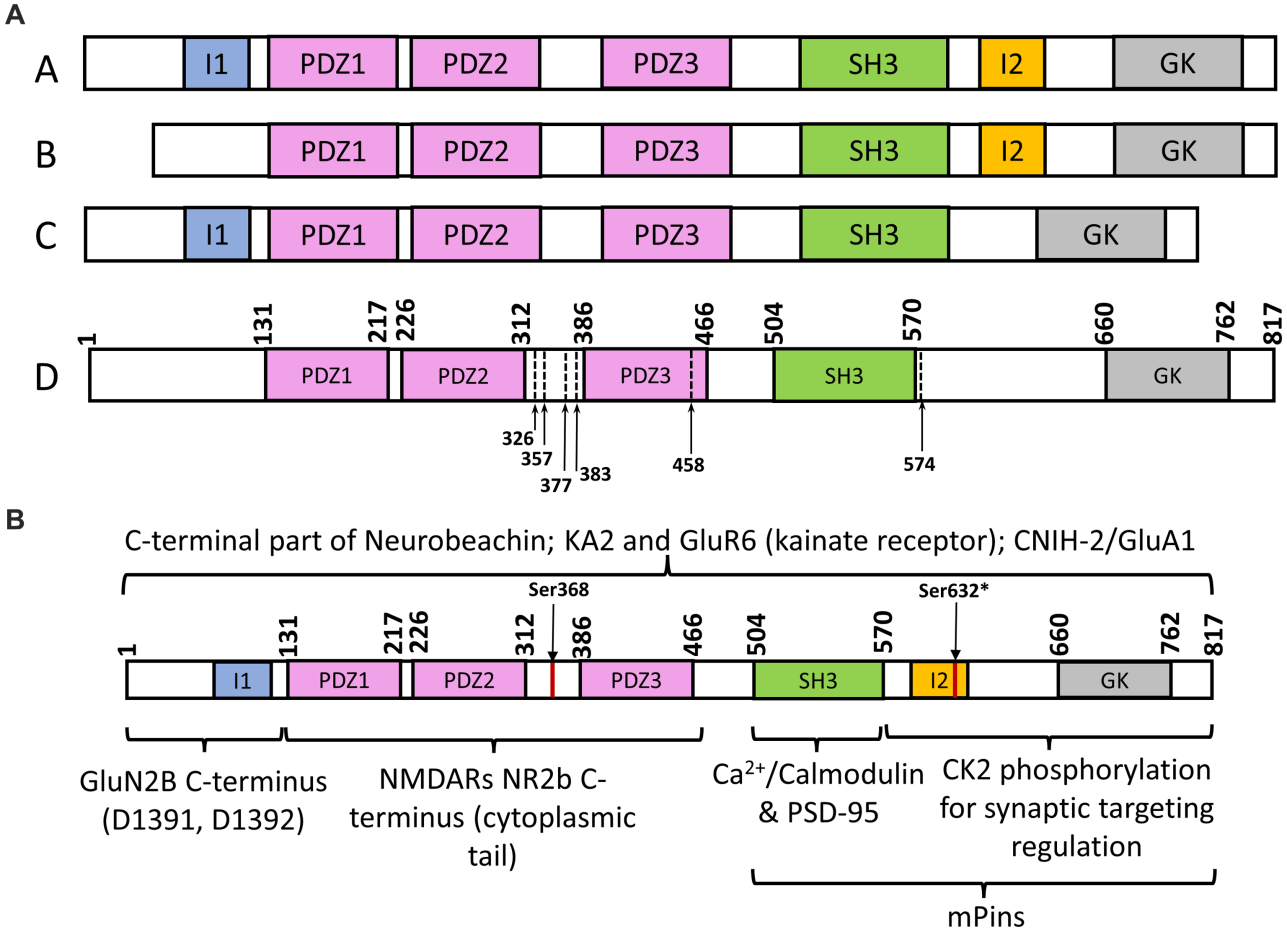
**(A)** Schematic representation of SAP102 splice variants and mutations. (A) SAP102 (rat) variant with all functional domains; (B) SAP102 (rat) splice variant with missing I1 region; (C) SAP102 (rat) splice variant with missing I2 region; (D) SAP102 (human) with identified truncations of mutants, stops codons within or before third PDZ domain. **(B)** Schematic representation of SAP102 (*Ser632 is for rat SAP102 sequence; I1 and I2 domains are in rat SAP102, not human SAP102) interaction with different targets, including Neurobeachin (Lauks et al., 2012), KA2 and GluR6 (kainate receptor; Garcia et al., 1998), CNIH-2/GluA1 (Liu et al., 2018), GluNR2B (NMDARs; Lau et al., 1996; Müller et al., 1996; Chen et al., 2011), Ca^2+^/Calmodulin/PSD-95 (Masuko et al., 1999), casein kinase II (CK2; Wei et al., 2018); mPins (Sans et al., 2005).

SAP102 is a scaffolding protein encoded by the disc-large homolog 3 (*DLG3*) gene and primarily functions in regulating synaptic trafficking of glutamate receptors and postsynaptic plasticity. Unlike PSD-95, it is most prevalent in the early stage of synapse development (Figure 1A; Oliva et al., 2012). About 80% of spine-localized SAP102 is mobile, whereas the mobility is observed in only 36% of the PSD-95 within spines (Zheng et al., 2010). SAP102 shares structural similarities with other MAGUKs. It consists of three tandem PDZ domains at the amino-terminus followed by an src homology (SH3) domain and a carboxy-terminal guanylate kinase (GK) domain. Compared with other MAGUK homologs, the amino-terminus of SAP102 is considerably longer and more flexible, and has a unique cysteine- and histidine-rich region (Firestein et al., 2000). SAP102 (rat) has three naturally occurring N-terminal and C-terminal splice variants (Figure 2A), including an 18-amino acid deletion of the I1 region close to the PDZ1 domain (variant B in Figure 2A) and a 14-amino acid deletion of the connecting loop between SH3 and GK domains (I2 region, variant C in Figure 2A; Müller et al., 1996). Interestingly, the I1 region is recognized by the GluN2B subunit, and this interaction promotes the lengthening of dendritic spines and the formation of synapses (Chen et al., 2011). The primary function of SAP102 is to regulate the trafficking and scaffolding of α-amino-3-hydroxy-5-methyl-4-isoxazolepropionic acid (AMPA) receptors and N-methyl-D-aspartate (NMDA) glutamate receptors (Figure 1B). However, the mechanisms by which it accomplishes its role, particularly during the early development of neurons, remain unknown. Given the importance of SAP102 in the early stage of neuronal development, understanding its functional roles in detail during synaptogenesis would be the key for developing new therapeutic strategies for neuroregeneration. In this review, we focus on synaptic functions of SAP102, regulatory mechanisms of SAP102 functions, especially its expression and functions in the early stage of synaptogenesis, and SAP102-linked neurodevelopmental disorders.

## Synaptic functions of SAP102

MAGUKs are known to scaffold and cluster ionotropic excitatory glutamate receptors, including AMPA receptors (AMPARs), NMDA receptors (NMDARs), and kainate receptors. SAP102 is mostly associated with AMPARs and NMDARs, but the process of how it scaffolds and regulates these receptors remains obscure. The PDZ domains of SAP102 have been shown to interact with the (E)S/TXV/I/L recognition motif in the cytosolic carboxy-terminus of the NR2 subunits of the NMDARs, enabling SAP102 activity in clustering and targeting NMDARs in the postsynaptic density (Figures 1B, 2B; Lau et al., 1996; Müller et al., 1996). More specifically, SAP102 functions during the early stage of NMDAR processing through its first two PDZ domains by interacting with Sec8 (Sans et al., 2003). To study NMDAR trafficking, the interactions between PDZ domains and an exocyst complex were observed. The protein Sec8 from the exocyst complex interacted strongly with PDZ1 and PDZ2 but interacted to a lesser degree with PDZ3. A mutant of Sec8 lacking the last four amino acids did not interact with any of the three PDZ domains. Co-immunoprecipitation experimental results indicates that a molecular complex containing SAP102, NR2B, and Sec8 forms in the endoplasmic reticulum (Figure 1B; Sans et al., 2003). This is also supported by the evidence that SAP102 binds to the NR2B subunit of NMDARs (Chen et al., 2011). A correlation between dendritic spine lengths and SAP102 splice variants was discovered when SAP102 was found to promote the formation of long spines. Specifically, SAP102 I1 promoted an increased formation of dendritic spines of all lengths (Chen et al., 2011). Further insight into SAP102’s interaction with the NR2B subunit of NMDAR identified two key residues, D1391 and D1392 (Figure 2B). It was found that mutations of these two residues hindered the PDZ-independent binding to SAP102. However, more research is needed to show whether these two key residues represent a direct binding site or the mutations result in disruptive conformational changes (Chen et al., 2012). The synaptic functions of SAP102 in regulating the trafficking and targeting of glutamate receptors are modulated by mPins (Figure 1B; Sans et al., 2005). By direct interaction, mPins promotes SAP102 SH3/GK domains to form a complex with the NMDAR to induce the receptor clustering while also influencing its surface expression (Sans et al., 2005).

Regarding AMPARs, a study focused on both SAP102 and PSD-95 found that only the interaction of SAP102 with AMPARs was dependent on cornichon-2 (CNIH-2), an auxiliary subunit of AMPARs (Figure 1B; Liu et al., 2018). It suggests that SAP102 and PSD-95 regulate different AMPAR complexes. Interestingly, CNIH-2 does not have a PDZ ligand; therefore, it is highly unlikely that CNIH-2 interacts with SAP102 directly via PDZ domains, and also encourages future research to identify if the interaction is a multistep process, explain the detailed multistep process of MAGUK family proteins and their interactions with the receptors, and indicate the AMPAR auxiliary subunit associated with each MAGUK protein (Liu et al., 2018). In addition, SAP102 was shown to interact with the kainate receptor subtypes, KA2 and GluR6, which was demonstrated by the coimmunoprecipitation of SAP102 from brain extracts with both KA2 and GluR6 (Figures 1B, 2B; Garcia et al., 1998). However, it is obscure which domain (s) of SAP102 may contribute to the binding. Finally, SAP102 was also identified as a Neurobeachin (Nbea) binding protein in mouse brain by mass spectrometry (Lauks et al., 2012). It interacts with the C-terminal part of Nbea, which includes four domains (DUF, PH, BEACH, and WD40; Lauks et al., 2012). Nbea is highly expressed and required for the development and functioning of central and neuromuscular synapses (Olszewski et al., 2012). Nbea plays an important role in trafficking membrane proteins to the pre- and post-synaptic sites; however, the precise mechanism and function of SAP102 binding with Nbea in the synapses is also under the veil (Lauks et al., 2012).

## Structural and functional regulation of SAP102 by various mechanisms

Phosphorylation is an important cellular regulatory mechanism in which a negatively charged phosphate group is transferred from phosphate-donating molecules to specified substrates or functional groups of a protein; this alters protein conformation and/or interferes with a protein’s interactions with its targets (Walaas and Greengard, 1991). Previous studies showed that phosphorylation of PSD-95 at serine 73 triggers the destabilization of the postsynaptic density, leading to the inhibition of spine growth and synaptic plasticity (Steiner et al., 2008). PSD-95 S73 is a CaMKII consensus phosphorylation site that can be phosphorylated to regulate the association of PSD-95 with NMDARs (Steiner et al., 2008). Interestingly, the protein sequence alignment shows that the region containing Ser73 is highly conserved in both PSD-95 and SAP102. It is likely that the same serine in SAP102 can be phosphorylated as a means of functional regulation. In fact, SAP102 synaptic targeting and mobility is regulated by C-terminal splicing and phosphorylation (Wei et al., 2015, 2018). Studies have shown that SAP102 splice variants that contain a C-terminal I2 domain (Figure 2A) are found abundantly at dendritic spines and the expression of the I2 splicing isoforms are modulated developmentally, indicating alternative splicing regulates synaptic localization of SAP102 (Wei et al., 2015). Furthermore, phosphorylation of the Ser632 (rat SAP102) in the I2 domain by casein kinase II (CK2) increases the synaptic enrichment of SAP102 while also decreasing its mobility in dendritic spines (Wei et al., 2018). Similarly, Ser368 of SAP102 (human) can be phosphorylated by the c-Jun N-terminal kinases (JNKs) to affect the dynamics of SAP102, and this modification by JNK is restricted to neurons that harbor mature synapses (Kunde et al., 2021). These results suggest that phosphorylation plays a key role in regulating SAP102 synaptic targeting and mobility.

Lipid modification, particularly palmitoylation, is an important post-translational mechanism contributing to the distribution and membrane targeting of PSD-MAGUKs at synapses. Postsynaptic targeting of PSD-95 requires palmitoylation of two cysteine residues (Cys3 and Cys5) at its amino-terminal end (Firestein et al., 2000). In addition, PSD-95 postsynaptic membrane targeting induced by palmitoylation is regulated by calmodulin (CaM) in the presence of Ca^2+^ (Zhang et al., 2014). Unlike other MAGUK members, the N-terminal cysteines of SAP102 are not palmitoylated (Firestein et al., 2000), which may lead to SAP102’s increased synaptic mobility. This is supported by data showing that SAP102 has been found to play a role in axonal targeting (El-Husseini et al., 2000). Not owing to palmitoylation, SAP102 targeting postsynaptic sites is induced by a unique postsynaptic targeting motif within the N-terminal domain (Firestein et al., 2000).

Moreover, SAP102 is mediated by zinc and calcium. Among the MAGUKs, SAP102 has a unique and extra-long N-terminus containing a cysteine- and histidine-rich motif. Studies found that the cysteines at the N-terminus of SAP102 tightly bind to zinc, and adding ZnCl_2_ into the solution of the N-terminal peptide induces the disordered apo-peptide to form a secondary structure (El-Husseini et al., 2000). Recently, zinc was identified as a novel modulator of PSD-95 postsynaptic membrane association by chelating its amino-terminal region, thus impairing its ability of palmitoylation (Zhang et al., 2021). The binding of zinc to SAP102’s N-terminus is thought to regulate synaptic vesicle trafficking and fusion, but the mechanistic details remain unknown. SAP102 can bind CaM in a calcium-dependent manner via its SH3 region to interact with PSD-95 (Figure 2B), the CaM-binding site in SAP102 is independent of the interaction between SAP102 and the C-terminus of the NR2B subunit (Masuko et al., 1999). These results suggest that Ca^2+^/CaM modulates the interaction of SAP102 with other MAGUKs and regulates their functions at synapses.

## Functional roles of SAP102 as a highly mobile PSD-MAGUK in the early stage of synaptic development

It is well-known that MAGUKs can be found presynaptically and postsynaptically during development in the brain. However, studies have shown that these proteins exhibit differences in their temporal and spatial expression. PSD-95 is mostly restricted to the postsynaptic density with a low expression during embryonic and early postnatal development brain regions, but the expression is enhanced during postnatal development and reaches a maximum level in adults (Figure 1A; Oliva et al., 2012). In contrast, SAP102 is enriched in hippocampus during the first postnatal week, maintains a high expression by postnatal day P35 and then decreases in the adult stage (Figure 1A; Müller et al., 1996; Oliva et al., 2012; Sans et al., 2000). The unique expression pattern of SAP102 reflects its specific function in the early stage of synaptogenesis.

SAP102 is a highly mobile MAGUK. Many studies have suggested that it is expressed early and dominates at immature synapses whilst the expression of PSD95 mainly occurs in the mature neurons (Wei et al., 2018). When analyzing dendritic architecture, it was found that immature neuron cultures strongly correlated with the presence of NMDARs expressing the NR2B subunit (Bustos et al., 2014). As these neural cultures matured, the levels of NMDAR expressing NR2B declined, whereas those that expressed NR2A increased (Bustos et al., 2014). This difference in NMDAR subunit composition has substantial implications on synaptic architecture and development as the synaptic trafficking of these distinct glutamate receptor subtypes is mediated differentially by individual MAGUK members at different developmental stages (Bustos et al., 2014). PSD95 can only interact with NR2A-NMDARs, while SAP102 can interact with both NR2A and NR2B-NMDARs (Elias et al., 2008). It is believed that the reason why SAP102 expression dominates in the younger neurons is due to the abundance of NR2B-NMDARs relative to NR2A. However, the exact mechanisms have not been found yet (Elias et al., 2008). Previous studies demonstrated that SAP102 regulates cortical synapse development through the ephrin/EphB and p21-activated protein kinase (PAK) signaling pathways (Murata and Constantine-Paton, 2013). The Eph signaling pathway is an important cell communication tool that performs a leading role in embryonic development. SAP102 was revealed to form a complex with the receptor tyrosine kinase EphB2 and RacGEF Kalirin-7 in the early postnatal cortex, and SAP102 knockdown reduced the surface and dendritic expression of EphB2 and prevented reorganization of actin filaments and synapse formation through negatively affecting the EphB signaling pathway (Murata and Constantine-Paton, 2013). The PAK regulates diverse cellular functions, including cytoskeletal actin assembly. It was discovered that PAK kinase activity was downregulated by SAP102 knockdown (Murata and Constantine-Paton, 2013). However, the structural and molecular mechanisms of SAP102 mediating EphB and PAK is also elusive. More recently, a study showed that SAP102 expression is modulated by Smad4, a target of miR-431 (Ge et al., 2023). We can define the detailed molecular interactions that drive synaptogenesis by further elucidating the interplay between MAGUK proteins and other essential neural proteins such as the NMDARs.

## Mutations and changes in SAP102 expression causing neurological disorders

Several studies have shown that SAP102 missense variants are associated with neurological disorders. Truncated SAP102 proteins (premature stop codons before or within the third PDZ domain) were found to cause moderate to severe X-linked intellectual disability (XLID) and other intellectual impairments (Tarpey et al., 2004). Gene analysis revealed the presence of a stop codon at amino acid position 326, 377, 383, and 458 of the *DLG3* gene coded SAP102 (variant D in Figure 2A), removing part of the normally translated protein. These gene variants were identified from families with XLID by automated high-throughput mutation detection screening methods (Tarpey et al., 2004). Studies on murine models have further demonstrated that SAP102 mutant mice developed impairments in hippocampus-dependent and NMDAR-dependent spatial tasks (Cuthbert et al., 2007). A splice site mutation (IVS6-1G > A) in the SAP102 gene was identified in one out of 300 families with moderate to severe non-syndromic intellectual disability, the first intellectual disability gene directly linked to glutamate receptor signaling and trafficking (Zanni et al., 2010). This genetic mutation introduced a translational frameshift, resulting in termination at position 357 (Pro) between the second and third PDZ domain of SAP102 (Zanni et al., 2010). Another nonsense variation in the DLG3 gene was reported from a family with XLID (Sandestig et al., 2020). Here, a single nucleotide substitution (c.1720C > T) leads to a stop codon at Arg574 and consequently premature truncation of the protein (Sandestig et al., 2020). Alternations in SAP102 genetic sequence can modify synaptic function early in development resulting in changes in network connectivity later in life. It was proven after determining that the effect of a genetic deletion of SAP102 led to a decrease in the total number of thalamocortical (TC) axons innervating the somatosensory cortex (Crocker-Buque et al., 2016).

Aside from the *DLG3* gene mutations, abnormal expression of SAP102 in the hippocampus also contributes to the development of neurological diseases. In a study focused on the relation of NMDAR interacting PSD proteins with bipolar disorder, major depression, and schizophrenia, a decreased expression of SAP102 was observed in all three psychiatric disorders, suggesting that a lack of SAP102 is linked to the development of mental illnesses (Kristiansen and Meador-Woodruff, 2005). To study Alzheimer’s disease (AD), the leading form of dementia, Wistar rats and APP/PS1 mice (AD mouse model) were used to investigate the dynamic SAP102 expression in the hippocampal subregion at various ages (Su et al., 2018). In the APP/PS1 mice, the results demonstrated that the alternations in SAP102 expression in CA1 and CA3 hippocampal regions served as early responsive markers in the prodromal disease stage since these regions are early responsive area. However, the SAP102 expression became significantly lower than that in WT mice in DG and CA3, suggesting the changes were more sensitive in these subfields as the DG and CA3 are more vulnerable and prone to both impairment and disease progression (Su et al., 2018). Wistar rats showed that SAP102 was highly expressed in the postnatal stages and adulthood, indicating that SAP102 may still have an important function in later stages of life. This was a surprising discovery because it was previously known that SAP102 is primarily dominant in earlier stages of life and then is replaced by PSD-95 in adulthood (Su et al., 2018). Moreover, significantly reduced protein levels of SAP102 were found in the AD brain, indicative of the importance of SAP102 in AD progression (Proctor et al., 2010). These results reflect that SAP102 expression level is highly related to variable neurological diseases.

## Concluding remarks

Neurodevelopmental and neurodegenerative disorders such as Alzheimer’s or Parkinson’s diseases are tightly linked to structural loss of synapses and synaptic dysfunction. These diseases have been described for over 100 years yet are still uncurable. Extensive efforts have focused on searching for novel, efficient neuroprotectors and therapeutic strategies to prevent the development of neurodegenerative progress. Unfortunately, these efforts are hampered due to limited knowledge about the detailed mechanisms driving neurodegeneration. SAP102 is characterized as a highly mobile synaptic MAGUK prevalent in the early stage of excitatory synapse formation. However, its synaptic functions during synaptogenesis remain insufficiently understood. SAP102 has an extra-long N-terminus with an I1 domain and multiple cysteine and histidine residues, yet it does not undergo palmitoylation, which is unique compared to the other homologs. The N-terminal domain with the alternatively spliced I1 region has been shown to play critical roles in controlling NMDAR interaction and spine morphology during synaptogenesis. It is also likely related to the high synaptic mobility of SAP102, but the structural and molecular mechanisms are still elusive. Future efforts may include studies on SAP102’s interactions with glutamate receptors to build up comprehensive and dynamic pictures of how SAP102 is involved in the trafficking and targeting of these receptors. For example, uncovering the precise molecular steps of CNIH-2-dependent AMPAR regulation by SAP102 and the structural and molecular details of different domains of SAP102 working cooperatively to interact with NMADRs highlight unique SAP102-dependent roles in synaptic functions. Moreover, the identification of new molecular targets will be fundamental for constructing a SAP102-interaction molecular network. Considering the importance of PSD-MAGUK proteins in many neurological disorders, it would be extremely valuable to better understand their structures, functions, and regulatory mechanisms at the synapse to identify therapeutic targets to prevent the development of neurodegenerative progress.

Another potential therapeutic strategy may be neuroregeneration including regrowth or repair of the nervous tissue to compensate for structural and functional loss of synapses. Precise functions and molecular mechanisms of SAP102 in the early stages of neural development and how SAP102 gene expression is regulated would be further research topics to explore, especially identifying transcription factors that regulate SAP102 gene expression during synaptogenesis. More studies are also needed to characterize the effect of the pathogenic *DLG3* variants on synaptic function. For example, it would be interesting to utilize the missense variants as disease models to investigate whether they induce structural changes and exert their effects indirectly through the SAP102 binding partners mentioned above. Ultimately, it would be highly relevant to evaluate whether an increase in SAP102 expression levels through transcription promotion could alleviate some of the symptoms of individuals with *DLG*3 variants.

## Author contributions

DAD: Writing – original draft, Writing – review & editing. MYK: Writing – original draft, Writing – review & editing. YZ: Conceptualization, Formal Analysis, Project administration, Supervision, Validation, Visualization, Writing – original draft, Writing – review & editing.

## References

[ref1] Bustos F. J. Varela-Nallar L. Campos M. Henriquez B. Phillips M. Opazo C.. (2014). PSD95 suppresses dendritic arbor development in mature hippocampal neurons by occluding the clustering of NR2B-NMDA receptors. PLoS One 9:e94037. doi: 10.1371/journal.pone.0094037, PMID: 24705401 PMC3976375

[ref2] Chen B. S. Gray J. A. Sanz-Clemente A. Wei Z. Thomas E. V. Nicoll R. A.. (2012). SAP102 mediates synaptic clearance of NMDA receptors. Cell Rep. 2, 1120–1128. doi: 10.1016/j.celrep.2012.09.024, PMID: 23103165 PMC3513525

[ref3] Chen B. S. Thomas E. V. Sanz-Clemente A. Roche K. W. (2011). NMDA receptor-dependent regulation of dendritic spine morphology by SAP102 splice variants. J. Neurosci. 31, 89–96. doi: 10.1523/JNEUROSCI.1034-10.2011, PMID: 21209193 PMC3030119

[ref4] Crocker-Buque A. Currie S. P. Luz L. L. Grant S. G. Duffy K. R. Kind P. C.. (2016). Altered thalamocortical development in the SAP102 knockout model of intellectual disability. Hum. Mol. Genet. 25, 4052–4061. doi: 10.1093/hmg/ddw244, PMID: 27466188 PMC5291236

[ref5] Cuthbert P. C. Stanford L. E. Coba M. P. Ainge J. A. Fink A. E. Opazo P.. (2007). Synapse-associated protein 102/dlgh3 couples the NMDA receptor to specific plasticity pathways and learning strategies. J. Neurosci. 27, 2673–2682. doi: 10.1523/JNEUROSCI.4457-06.2007, PMID: 17344405 PMC2851144

[ref6] el-Husseini A. E. Topinka J. R. Lehrer-Graiwer J. E. Firestein B. L. Craven S. E. Aoki C.. (2000). Ion channel clustering by membrane-associated guanylate kinases. Differential regulation by N-terminal lipid and metal binding motifs. J. Biol. Chem. 275, 23904–23910. doi: 10.1074/jbc.M909919199 10779526

[ref7] Elias G. M. Elias L. A. B. Apostolides P. F. Kriegstein A. R. Nicoll R. A. (2008). Differential trafficking of AMPA and NMDA receptors by SAP102 and PSD-95 underlies synapse development. Proc. Natl. Acad. Sci. U. S. A. 105, 20953–20958. doi: 10.1073/pnas.0811025106, PMID: 19104036 PMC2634944

[ref8] Firestein B. L. Craven S. E. Bredt D. S. (2000). Postsynaptic targeting of MAGUKs mediated by distinct N-terminal domains. Neuroreport 11, 3479–3484. doi: 10.1097/00001756-200011090-00016, PMID: 11095503

[ref9] Garcia E. P. Mehta S. Blair L. A. C. Wells D. G. Shang J. Fukushima T.. (1998). SAP90 binds and clusters kainate receptors causing incomplete desensitization. Neuron 21, 727–739. doi: 10.1016/S0896-6273(00)80590-5, PMID: 9808460

[ref10] Ge J. Xue Z. Shu S. Yu L. Qin R. Tao W.. (2023). MiR-431 attenuates synaptic plasticity and memory deficits in APPswe/PS1dE9 mice. JCI Insight 8:e166270. doi: 10.1172/jci.insight.166270, PMID: 37192007 PMC10371242

[ref11] Kristiansen L. V. Meador-Woodruff J. H. (2005). Abnormal striatal expression of transcripts encoding NMDA interacting PSD proteins in schizophrenia, bipolar disorder and major depression. Schizophr. Res. 78, 87–93. doi: 10.1016/j.schres.2005.06.012, PMID: 16023328

[ref12] Kunde S.-A. Schmer B. Ahmadyar E. Rademacher N. Zieger H. L. Shoichet S. A. (2021). JNK activity modulates postsynaptic scaffold protein SAP102 and kainate receptor dynamics in dendritic spines. bioRxiv:2021.04.30.442109. doi: 10.1101/2021.04.30.442109 PMC11081805 38582451

[ref13] Lau L. F. Mammen A. Ehlers M. D. Kindler S. Chung W. J. Garner C. C.. (1996). Interaction of the N-methyl-D-aspartate receptor complex with a novel synapse-associated protein, SAP102. J. Biol. Chem. 271, 21622–21628. doi: 10.1074/jbc.271.35.21622, PMID: 8702950

[ref14] Lauks J. Klemmer P. Farzana F. Karupothula R. Zalm R. Cooke N. E.. (2012). Synapse associated protein 102 (SAP102) binds the C-terminal part of the scaffolding protein neurobeachin. PLoS One 7:e39420. doi: 10.1371/journal.pone.0039420, PMID: 22745750 PMC3380004

[ref15] Levy A. M. Gomez-Puertas P. Tümer Z. (2022). Neurodevelopmental disorders associated with PSD-95 and its interaction partners. Int. J. Mol. Sci. 23:4390. doi: 10.3390/ijms23084390, PMID: 35457207 PMC9025546

[ref16] Liu M. Shi R. Hwang H. Han K. S. Wong M. H. Ren X.. (2018). SAP102 regulates synaptic AMPAR function through a CNIH-2-dependent mechanism. J. Neurophysiol. 120, 1578–1586. doi: 10.1152/jn.00731.2017, PMID: 30067114 PMC6230800

[ref17] Masuko N. Makino K. Kuwahara H. Fukunaga K. Sudo T. Araki N.. (1999). Interaction of NE-dlg/SAP102, a neuronal and endocrine tissue-specific membrane-associated guanylate kinase protein, with calmodulin and PSD-95/SAP90. A possible regulatory role in molecular clustering at synaptic sites. J. Biol. Chem. 274, 5782–5790. doi: 10.1074/jbc.274.9.5782, PMID: 10026200

[ref18] Müller B. M. Kistner U. Kindler S. Chung W. J. Kuhlendahl S. Fenster S. D.. (1996). SAP102, a novel postsynaptic protein that interacts with NMDA receptor complexes in vivo. Neuron 17, 255–265. doi: 10.1016/S0896-6273(00)80157-9, PMID: 8780649

[ref19] Murata Y. Constantine-Paton M. (2013). Postsynaptic density scaffold SAP102 regulates cortical synapse development through EphB and PAK signaling pathway. J. Neurosci. 33, 5040–5052. doi: 10.1523/JNEUROSCI.2896-12.2013, PMID: 23486974 PMC3632365

[ref20] Oliva C. Escobedo P. Astorga C. Molina C. Sierralta J. (2012). Role of the MAGUK protein family in synapse formation and function. Dev. Neurobiol. 72, 57–72. doi: 10.1002/dneu.20949, PMID: 21739617

[ref21] Olszewski P. K. Rozman J. Jacobsson J. A. Rathkolb B. Strömberg S. Hans W.. (2012). Neurobeachin, a regulator of synaptic protein targeting, is associated with body fat mass and feeding behavior in mice and body-mass index in humans. PLoS Genet. 8:e1002568. doi: 10.1371/journal.pgen.1002568, PMID: 22438821 PMC3305408

[ref22] Proctor D. T. Coulson E. J. Dodd P. R. (2010). Reduction in post-synaptic scaffolding PSD-95 and SAP-102 protein levels in the Alzheimer inferior temporal cortex is correlated with disease pathology. J. Alzheimers Dis. 21, 795–811. doi: 10.3233/JAD-2010-100090, PMID: 20634587

[ref23] Sandestig A. Green A. Aronsson J. Ellnebo K. Stefanova M. (2020). A novel DLG3 mutation expanding the phenotype of X-linked intellectual disability caused by DLG3 nonsense variants. Mol. Syndromol. 10, 281–285. doi: 10.1159/000502601, PMID: 32021600 PMC6997794

[ref24] Sans N. Petralia R. S. Wang Y. X. Blahos J. II Hell J. W. Wenthold R. J. (2000). A developmental change in NMDA receptor-associated proteins at hippocampal synapses. J. Neurosci. 20, 1260–1271. doi: 10.1523/JNEUROSCI.20-03-01260.2000, PMID: 10648730 PMC6774158

[ref25] Sans N. Prybylowski K. Petralia R. S. Chang K. Wang Y. X. Racca C.. (2003). NMDA receptor trafficking through an interaction between PDZ proteins and the exocyst complex. Nat. Cell Biol. 5, 520–530. doi: 10.1038/ncb990, PMID: 12738960

[ref26] Sans N. Wang P. Y. du Q. Petralia R. S. Wang Y. X. Nakka S.. (2005). mPins modulates PSD-95 and SAP102 trafficking and influences NMDA receptor surface expression. Nat. Cell Biol. 7, 1179–1190. doi: 10.1038/ncb1325, PMID: 16299499

[ref27] Steiner P. Higley M. J. Xu W. Czervionke B. L. Malenka R. C. Sabatini B. L. (2008). Destabilization of the postsynaptic density by PSD-95 serine 73 phosphorylation inhibits spine growth and synaptic plasticity. Neuron 60, 788–802. doi: 10.1016/j.neuron.2008.10.014, PMID: 19081375 PMC2671083

[ref28] Su D. Liu H. Liu T. Zhang X. Yang W. Song Y.. (2018). Dynamic SAP102 expression in the hippocampal subregions of rats and APP/PS1 mice of various ages. J. Anat. 232, 987–996. doi: 10.1111/joa.12807, PMID: 29574717 PMC5979819

[ref29] Tarpey P. Parnau J. Blow M. Woffendin H. Bignell G. Cox C.. (2004). Mutations in the DLG3 gene cause nonsyndromic X-linked mental retardation. Am. J. Hum. Genet. 75, 318–324. doi: 10.1086/422703, PMID: 15185169 PMC1216066

[ref30] Vyas Y. Montgomery J. M. (2016). The role of postsynaptic density proteins in neural degeneration and regeneration. Neural Regen. Res. 11, 906–907. doi: 10.4103/1673-5374.184481, PMID: 27482211 PMC4962580

[ref31] Walaas S. I. Greengard P. (1991). Protein phosphorylation and neuronal function. Pharmacol. Rev. 43, 299–349. PMID: 1956954

[ref32] Wei Z. Behrman B. Wu W. H. Chen B. S. (2015). Subunit-specific regulation of N-methyl-D-aspartate (NMDA) receptor trafficking by SAP102 protein splice variants. J. Biol. Chem. 290, 5105–5116. doi: 10.1074/jbc.M114.599969, PMID: 25555912 PMC4335245

[ref33] Wei Z. Wu G. Chen B. S. (2018). Regulation of SAP102 synaptic targeting by phosphorylation. Mol. Neurobiol. 55, 6215–6226. doi: 10.1007/s12035-017-0836-4, PMID: 29282697 PMC6599631

[ref34] Won S. Levy J. M. Nicoll R. A. Roche K. W. (2017). MAGUKs: multifaceted synaptic organizers. Curr. Opin. Neurobiol. 43, 94–101. doi: 10.1016/j.conb.2017.01.006, PMID: 28236779 PMC5447471

[ref35] Zanni G. van Esch H. Bensalem A. Saillour Y. Poirier K. Castelnau L.. (2010). A novel mutation in the DLG3 gene encoding the synapse-associated protein 102 (SAP102) causes non-syndromic mental retardation. Neurogenetics 11, 251–255. doi: 10.1007/s10048-009-0224-y, PMID: 19795139

[ref36] Zhang Y. Fang X. Ascota L. Li L. Guerra L. Vega A.. (2021). Zinc-chelating postsynaptic density-95 N-terminus impairs its palmitoyl modification. Protein Sci. 30, 2246–2257. doi: 10.1002/pro.4187, PMID: 34538002 PMC8521293

[ref37] Zhang Y. Matt L. Patriarchi T. Malik Z. A. Chowdhury D. Park D. K.. (2014). Capping of the N-terminus of PSD-95 by calmodulin triggers its postsynaptic release. EMBO J. 33, 1341–1353. doi: 10.1002/embj.201488126, PMID: 24705785 PMC4194123

[ref38] Zheng C.-Y. Petralia R. S. Wang Y. X. Kachar B. Wenthold R. J. (2010). SAP102 is a highly Mobile MAGUK in spines. J. Neurosci. 30, 4757–4766. doi: 10.1523/JNEUROSCI.6108-09.2010, PMID: 20357126 PMC2874826

[ref39] Zhou H. H. Tang Y. Zhang X. Y. Luo C. X. Gao L. Y. Wu H. Y.. (2015). Delayed Administration of tat-HA-NR2B9c promotes recovery after stroke in rats. Stroke 46, 1352–1358. doi: 10.1161/STROKEAHA.115.008886, PMID: 25851770

